# Effectiveness of Bleeding Control Methods in Rhinoplasty: A Systematic Review and Meta-Analysis

**DOI:** 10.1055/a-2706-1208

**Published:** 2026-01-30

**Authors:** Mohammad Reza Zamani, Behzad Imani, Rohollah Abbasi, Ashkan Karimi, Samad Moslehi

**Affiliations:** 1 Student Research Committee, Hamadan University of Medical Sciences, Hamadan, Iran; 2 Department of Operating Room, School of Paramedicine, Hamadan University of Medical Sciences, Hamadan, Iran; 3 Department of Otolaryngology, School of Medicine, Hamadan University of Medical Sciences, Hamadan, Iran; 4 Research Center for Health Sciences, Hamadan University of Medical Sciences, Hamadan, Iran

**Keywords:** rhinoplasty, septorhinoplasty, intraoperative bleeding, systematic review, meta-analysis

## Abstract

One of the most common complications of rhinoplasty and septorhinoplasty is intraoperative bleeding, which poses challenges for both surgeons and patients. This systematic review and meta-analysis aimed to evaluate the effectiveness of various bleeding control methods in rhinoplasty and septorhinoplasty surgeries.

This study conducted a systematic review and meta-analysis according to the Preferred Reporting Items for Systematic Reviews and Meta-Analyses (PRISMA) guidelines. A comprehensive search was performed in reputable international databases to identify relevant studies. Ultimately, 16 randomized controlled trials (RCTs) with 933 patients were included in the analysis. The bleeding control methods evaluated included tranexamic acid (TXA), desmopressin, steroids, magnesium sulfate, clonidine, remifentanil, and patient positioning (reverse Trendelenburg position). Data were combined using meta-analysis methods in STATA version 17, and the standardized mean difference (SMD) with 95% confidence intervals (CIs) was calculated to assess the effects of the methods. The results showed that TXA (SMD: −1.31; 95% CI: −2.01 to −0.62) and steroids (SMD: −1.07; 95% CI: −1.70 to −0.43) had the most significant impact on reducing bleeding. Patient positioning also showed a considerable effect (SMD: −0.65; 95% CI: −1.01 to −0.30), and desmopressin had a positive impact (SMD: −1.53; 95% CI: −3.12 to 0.06), though this effect was not statistically significant. This study demonstrates that pharmacological and non-pharmacological interventions, such as TXA and patient positioning, can significantly reduce intraoperative bleeding. However, further studies with larger sample sizes and standardized designs are recommended for magnesium sulfate, clonidine, and remifentanil methods.

Level of Evidence I.

## Introduction

Rhinoplasty is a facial plastic surgery that has gained significant popularity worldwide over the decades. The annual number of rhinoplasty procedures in the United States ranges from 250,000 to 300,000. 1 2 3 Like other surgeries, rhinoplasty is not without complications, with bleeding, edema, and ecchymosis being among the most common. These complications can cause significant physical and psychological stress for patients. A major contributor to these complications is osteotomy, which is performed during the procedure. 1 4 5 Excessive and uncontrolled bleeding can significantly prolong surgical time. 6 Rhinoplasty is performed in a confined space, and excessive intraoperative bleeding can reduce the surgeon's visibility, complicating the procedure. This may increase surgical and anesthesia time and heighten patient stress. 7 Although this type of surgery is not typically associated with severe bleeding, surgeons often require frequent suctioning to maintain a clear surgical field, which may increase the risk of damage to surrounding tissues. 8

The most common methods to reduce intraoperative bleeding include changing the patient's position, using medications such as antifibrinolytics, α- and β-adrenergic receptor blockers, steroids, desmopressin, controlled hypotension with intravenous remifentanil infusion, and topical vasoconstrictors. 9 10 11 12 Despite recent advances in surgical and anesthetic techniques, intraoperative bleeding remains an unresolved concern. 13 Although numerous reviews and meta-analyses have been conducted on the methods mentioned above, there is a lack of comprehensive studies that compare all these approaches.

This systematic review and meta-analysis aim to evaluate and compare the efficacy of seven different methods for controlling bleeding during rhinoplasty. By analyzing data from various studies, we strive to provide more substantial evidence on bleeding management strategies and help surgeons make better-informed decisions when selecting the most suitable methods to control bleeding.

## Methods

This study aimed to evaluate and assess the quality of bleeding control methods in patients undergoing rhinoplasty through a systematic review and meta-analysis, conducted following the Preferred Reporting Items for Systematic Reviews and Meta-Analyses (PRISMA) checklist. 14

### Search Strategy

The researchers searched six databases, PubMed, Scopus, Embase, Web of Science, Google Scholar, and the Cochrane Central Register of Controlled Trials (CENTRAL), from September to December 2023. The search included studies published from January 2000 to December 2023. The selected keywords for the international databases were “rhinoplasty,” “septorhinoplasty,” “cosmetic nasal surgery,” “intraoperative bleeding,” “hemostasis,” and “bleeding control.”

Search terms were combined using Boolean operators (AND, OR) and tailored for each database. The following search strategy was used in PubMed and adapted for other databases: (“rhinoplasty” OR “septorhinoplasty” OR “cosmetic nasal surgery”) AND (“intraoperative bleeding” OR “hemostasis” OR “bleeding control”).

The search was limited to title, abstract, and keywords. No language or time restrictions were applied. All retrieved data were imported into EndNote 21, and duplicate articles were automatically removed. Additionally, a manual reference check of the included studies was performed to identify any potentially relevant articles that might have been missed.

### Eligibility Criteria

Two authors (B.I. and A.K.) independently screened the search results. They selected studies relevant to the topic based on their titles or abstracts. After a thorough review of titles, abstracts, and, if necessary, full texts, any disagreements between the two authors were resolved through consensus or by consulting a third author (R.A.).

Only studies that explicitly investigated the effect of bleeding control methods in rhinoplasty were included in the analysis. Additionally, only RCTs were included in this study. The rationale for this selection was the high level of evidence generated by RCTs and their ability to reduce bias through randomization and control groups. Furthermore, some studies compared two drugs without a control group. Since including these studies alongside those comparing a drug to a control group could introduce methodological errors, we decided to include only studies where a drug was compared with a control group as the intervention. This approach enabled us to deliver high-quality and reliable results. Other inclusion criteria included reporting sample size and confidence intervals (CIs) in the study results. Non-English studies, review articles, and studies lacking a control group were excluded from the analysis.

### Risk-of-Bias Assessment

The risk of bias was assessed using the Cochrane guidelines for RCTs. The evaluation covered seven domains, including random sequence generation and allocation concealment (selection bias), blinding of participants and personnel (performance bias), blinding of outcome assessment (detection bias), incomplete outcome data (attrition bias), selective outcome reporting (reporting bias), and other potential sources of bias. Two review authors, M.R.Z. and A.K., independently assessed these domains. The risk of bias was categorized as low, unclear, or high, based on predefined criteria. 15 Additionally, a funnel plot based on the effect size of each study was used to assess publication bias. Furthermore, to quantitatively evaluate publication bias, we also employed Egger's test.

### Data Analysis

The mean and standard deviation for each treatment group and outcome were used to calculate the effect size. This study employed the standardized mean difference (SMD) as the measure of effect size. This method was chosen due to differences in measurement scales and units across studies. SMD standardizes mean differences based on a typical standard deviation, allowing for the comparison of study results obtained using different methods and measurement tools. This approach is particularly suitable when studies use heterogeneous scales to measure outcomes. Hedges' *g* was used to calculate SMD, which is adjusted for small sample sizes. A 95% CI was calculated for SMD to indicate the precision of effect estimates. A negative effect size indicates that the treatment methods were effective in reducing intraoperative bleeding compared with the control group. A forest plot was used to display effect sizes and 95% CIs. Additionally, a Galbraith plot was used to report study-specific effect sizes, their precision, and the overall effect size, as well as to identify outliers.

The analysis of treatment methods, including tranexamic acid (TXA), steroids, reverse Trendelenburg position (RTP), desmopressin, remifentanil, clonidine, and magnesium sulfate, was performed using STATA version 17 at a 5% significance level. In some studies, missing standard deviations for control and intervention groups were imputed using the method described by Idris and Robertson (2009). 16

### Assessment of Heterogeneity

Heterogeneity among studies was assessed using the *I* ^2^ statistic and the *Q* test. *I* ^2^ values were interpreted as 0 to 25% low heterogeneity, 25 to 50% moderate heterogeneity, 50 to 75% high heterogeneity, and >75% very high heterogeneity. Due to the presence of heterogeneity and differences in accurate effect sizes resulting from inherent variability among studies (e.g., differences in study populations or drug administration methods), a random-effects model was used. This model is more suitable for analyzing heterogeneity, as it accounts for variability between studies. Subgroup analysis was also performed to explore the sources of heterogeneity. If heterogeneity persisted within subgroups, they were further analyzed based on the drug administration methods.

## Results

### Study Selection

**Fig. 1:**
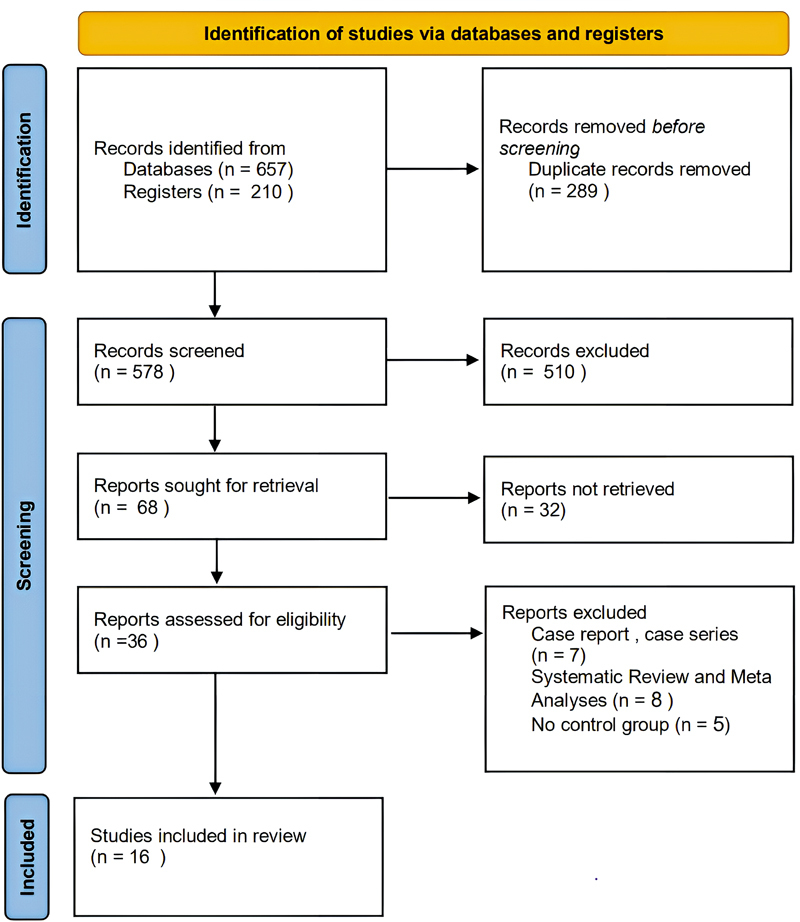
RISMA flow diagram illustrates the stages of article selection for this systematic review, from initial database search to final study inclusion, with numbers at each stage. PRISMA, Preferred Reporting Items for Systematic Reviews and Meta-Analyses.

Based on the search strategy, a total of 867 studies were identified. After removing 289 duplicate studies, 578 unique studies remained. Of these, 510 studies were excluded due to irrelevance to the study topic based on title and abstract screening. The full texts of 68 studies were retrieved, of which 32 were excluded due to the unavailability of full texts. Additionally, seven studies were excluded because they were case reports, and eight studies were excluded because they were systematic reviews or meta-analyses. Furthermore, five studies were excluded despite full-text review because they lacked a control group. 9 17 18 19 20 Ultimately, 16 studies (with 19 comparisons, as some studies had more than one relevant intervention group) met the eligibility criteria and were included in the meta-analysis. These studies evaluated the following methods: TXA, steroids, RTP, desmopressin, remifentanil, clonidine, and magnesium sulfate. 4 11 12 21 22 23 24 25 26 27 28 29 30 31 32 33 The flowchart of study selection and reasons for exclusion is presented in Fig. 1 .

### Characteristics of the Studies

This meta-analysis included 16 RCTs involving 933 patients, with 470 patients assigned to the control group. Studies excluded patients with hematological, endocrine, renal, or gastrointestinal diseases, as well as those using anticoagulant therapy. All studies included patients undergoing cosmetic rhinoplasty or septorhinoplasty. The studies varied in terms of intervention methods, patient populations, and measurement tools. The mean age of participants ranged from 23 to 58 years, and most studies included patients with a balanced gender distribution, with approximately 60 to 70% of participants being female. Only three studies did not report age and gender information. 12 22 32

The methods of drug administration included intravenous (IV), oral, and intranasal spray. The distribution and characteristics of the primary interventions are presented in a structured summary in Table 1 .

**Table 1:** Distribution of interventions by route and dosage

Intervention	Studies ( *n* )	Patients ( *n* )	Administration route	Dosage (number of studies)
Tranexamic acid	6	334	Intravenous	10 mg/kg (three studies)
			Oral	1 g (two studies)
			Topical	100 mg (one study)
Steroids	3	100	Intravenous	Dexamethasone: 8–30 mg/kg (two studies)
				Methylprednisolone: 1 mg/kg (one study)
Desmopressin	4	208	Intravenous	0.1 μg/kg (one study)
			Nasal spray	20–40 μg (three studies)
Position change	3	124	–	–

In the current analysis, control groups from the included studies were categorized into three main types:

1. Control groups receiving normal saline (six studies)
2. Control groups receiving unspecified placebo (three studies)
3. Control groups that did not receive the active intervention (five studies).

It should be noted that two studies provided no additional description of their control group protocol.

Two studies, Ozkose et al and Nooraei et al, used positional changes instead of pharmacological interventions. In the study by Ozkose et al, patients were divided into three groups, and the effects of positional changes on bleeding were evaluated. In the study by Nooraei et al, patients were divided into two groups: One in the RTP and the other in the head-up position. This study used the volume of suctioned blood and the weight of blood-soaked gauze as measures of bleeding. 12 31

In some studies, only the volume of blood suctioned was measured, while others also used the weight of blood-soaked gauze as an additional measure. Further details are provided in Table 2 .

**Table 2:** Characteristics of included studies in the meta-analysis

Study (year)	Number of patients	Mean age (years)/sex (male/female)	Administration	Intervention (drug/position)	Control group	Measurement tools
Beikaei et al (2015) 21	96	1. 25.9 ± 6.6 **/** 0/44 2. 26.0 ± 5.0 **/** 16/32	IV	1. TXA (10 mg/kg)	Normal saline	Suctioned blood volume + gauze weight
Haddady-Abianeh et al (2022) 23	42	1. 30.67 ± 8.27/8/34 2. 30.67 ± 8.27/8/34 3. 30.67 ± 8.27/8/34	1. IV 2. Spray	1. TXA (10 mg/kg) 2. DDAVP (40 μg)	Placebo	Suctioned blood volume
Haddady-Abianeh et al (2019) 22	30	–/–	Spray	DDAVP (40 μg)	Normal saline	Suctioned blood volume
Kosucu et al (2020) 24	49	1. 34.6 ± 10.4/10/15 2. 32.7 ± 13.36/11/1	IV	Magnesium sulfate (30–50 mg·kg 1 minute before induction of anesthesia and 10–20 mg·kg ^−1^ ·h − ^1^ by continuous IV infusion during surgery)	Normal saline	Suctioned blood volume
Ozkose et al (2016) 12	58	–/–	–	Position change	–	Suctioned blood volume
Eftekharian and Rajabzadeh (2016)	50	1. 24.72 ± 3.6/8/17 2. 22.32 ± 5.12/13/1	Oral	TXA (1 g)	Placebo	Suctioned blood volume + gauze weight
Akbarpour et al (2023) 26	120	1. 28.1/2/38 2. 26.9/2/38 3. 26.1/1/39	Spray	1. DDAVP (20 μg) 2. DDAVP (40 μg)	Normal saline	Suctioned blood volume
Youssefy et al (2022) 27	70	27 ± 10/33/37	IV	DDAVP (normal saline 500 mL, which consists of 0.1 μg/kg of desmopressin)	Normal saline	Suctioned blood volume + gauze weight
Sakallioglu et al (2015) 28	75	1. 28/13/12 2. 27/14/11 3. 29/15/10	Oral + IV	1. TXA (oral TXA as first dose 1 g starting 2 hours before surgery, 3 g daily in divided doses 1 g, every 8 hours for 5 days) 2. Methylprednisolone (a single dose of 1 mg/kg) 3. Placebo	N/A	Suctioned blood volume
Hazrati et al (2021) 11	60	1. 57.99 ± 0.566/12/18 2. 58 ± 0.564/12/18	Local	TXA (100 mg)	No intervention	Suctioned blood volume + gauze weight
Tuncel et al (2013) 29	60	29/28/32	IV	Dexamethasone (10–30 mg/kg)	No intervention	Suctioned blood volume
Tabrizi et al (2014) 30	66	1. 23.24 ± 4.12/11/2 2. 26.12 ± 6.06/17/1	Oral	Clonidine (0.2 mg)	Placebo	Suctioned blood volume + gauze weight
Ghavimi et al (2017) 4	50	28.78 ± 2.8/12/38	IV	TXA (10 mg/kg)	Normal saline	Suctioned blood volume + gauze weight
Nooraei et al (2012) 31	30	1. 26.73 ± 8.40/6/9 2. 27.27 ± 2.96/6/9	–	Reverse Trendelenburg position	No intervention	Suctioned blood volume + gauze weight
Kargi et al (2003) 32	60	–/–	IV	Dexamethasone (8–24 mg/kg)	No intervention	Suctioned blood volume
Kosucu et al (2014) 33	52	1. 24.5 ± 5.5/16/8 2. 24.4 ± 5.6/19/9	IV	Remifentanil (1 μg/kg)	No intervention	Suctioned blood volume

Abbreviations: IV, intravenous; TXA, tranexamic acid; DDAVP, desmopressin.

### Risk of Bias in Included Studies

**Fig. 2:**
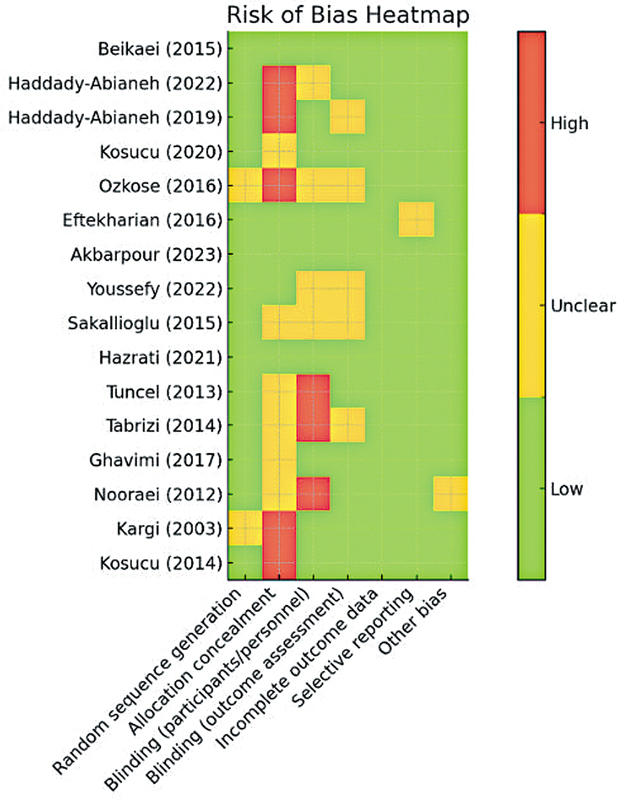
Heatmap showing risk of bias across studies. Green, low; yellow, unclear; red, high. Overall risk is shown in the last column.

The risk-of-bias assessment using the Cochrane Collaboration tool showed that most studies had a low risk of bias in the domains of random sequence generation and incomplete outcome data. Specifically, 14 out of 16 studies (87.5%) in the domain of random sequence generation and 15 out of 16 studies (93.7%) in the domain of incomplete outcome data had a low risk of bias. This indicates that randomization methods were appropriately performed, and outcome data were fully reported ( Fig. 2 ).

However, some studies had a high or unclear risk of bias in allocation concealment, blinding of participants and personnel, and blinding of outcome assessment. For example, six studies (37.5%) had an unclear risk of bias in allocation concealment, and four studies (25%) had a high risk of bias. In blinding participants and personnel, five studies (31.2%) had an unclear risk of bias, and three studies (18.7%) had a high risk of bias. This may be due to the lack of detailed reporting of blinding or allocation concealment methods in some studies. Most studies had a low risk of bias in terms of selective reporting and other potential sources of bias. Only one study (6.2%) in the selective reporting domain and two studies (12.5%) in the domain of other biases had an unclear risk of bias.

**Fig. 3:**
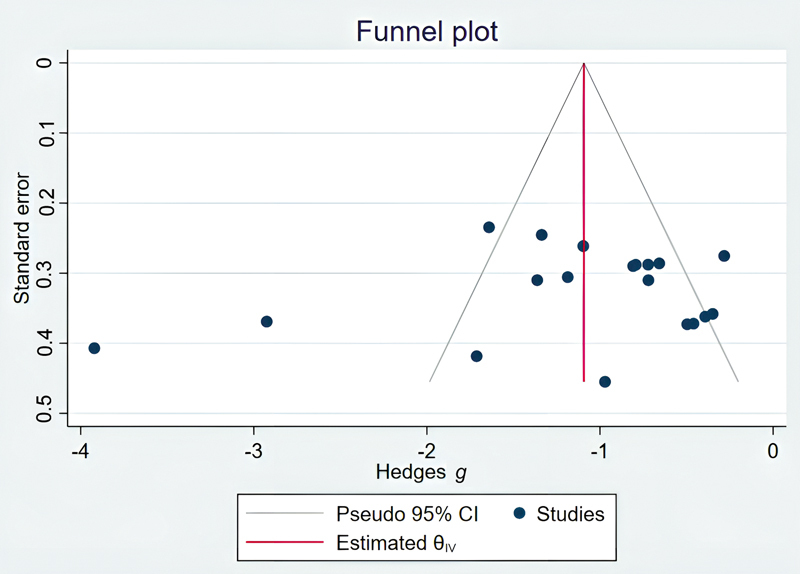
Funnel plots. Overall, no significant publication bias was observed; however, some small-scale studies require careful interpretation. CI, confidence interval.

The study by Ozkose et al (2016) 12 had an unclear or high risk of bias in the domains of random sequence generation, allocation concealment, blinding of participants and personnel, and blinding of outcome assessment. Additionally, the studies by Tuncel et al (2013) 29 and Tabrizi et al (2014) 30 had a high risk of bias due to inadequate blinding of participants and personnel. However, studies such as those by Beikaei et al (2015) 21 and Akbarpour et al (2023) 26 had a low risk of bias in all domains, indicating their high methodological quality. In addition, the funnel plot indicated a slight asymmetry in the distribution of studies, which may suggest the presence of partial publication bias ( Fig. 3 ). Furthermore, the results of Egger's test showed no significant publication bias ( *p* = 0.0526). However, since the number of studies in the subgroups was less than 10, it was not possible to conduct the test separately for each subgroup.

### Data Analysis and Treatment Subgroups

Out of the 16 RCTs included in this meta-analysis to determine the effect of methods on reducing bleeding during surgery, 463 patients receiving the mentioned methods for bleeding reduction were assigned to the treatment group, and 470 patients were assigned to the control group. The mean difference between the intervention and control groups across all studies is reported in Fig. 3 . The *Q* -value of 112.12 indicated heterogeneity among the study results ( *p* < 0.001).

**Fig. 4:**
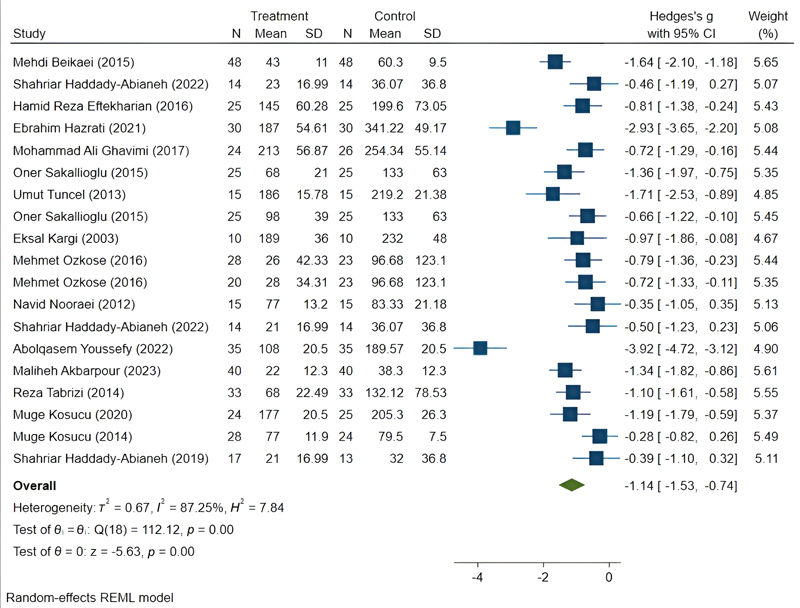
Pooled results of all bleeding control methods. CI, confidence interval; SD, standard deviation.

The SMD between the groups was −1.14 (95% CI: −1.53 to −0.74), indicating a significant association between treatment methods and reduced bleeding in cosmetic rhinoplasty. Considerable heterogeneity was observed among the studies ( *I* ^2^ = 87.25%; Fig. 4 ).

**Fig. 5:**
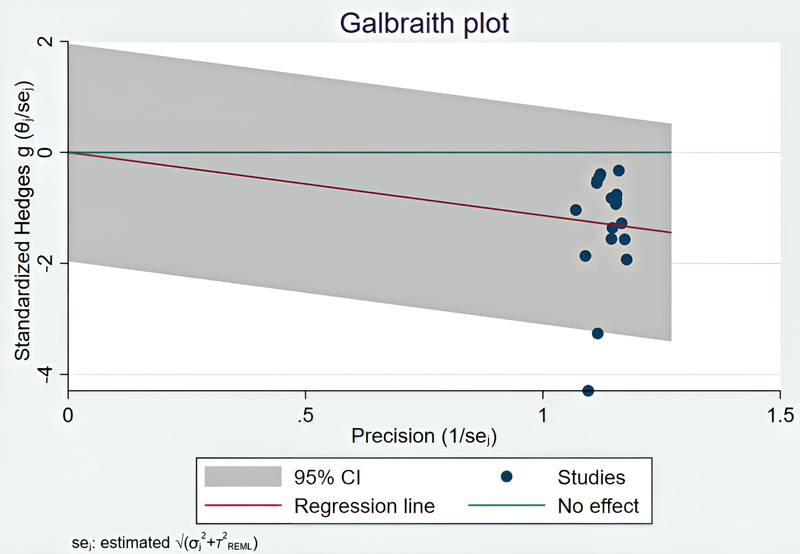
Galbraith plot of the effect sizes of treatments in reducing surgical bleeding during rhinoplasty and septorhinoplasty. The plot indicates that certain studies (e.g., Haddady-Abianeh et al [2022] ^23^ ; Hazrati et al [2021] ^11^ ) are major contributors to heterogeneity. CI, confidence interval.

The Galbraith plot is reported to assess heterogeneity in the effect size of the introduced treatments for reducing bleeding during cosmetic rhinoplasty ( Fig. 5 ).

Due to the high heterogeneity ( *I* ^2^ = 87.25%) among the studies included in this review, subgroup analyses were conducted to investigate the sources of heterogeneity and determine the effect of each treatment method on reducing surgical bleeding.

**Fig. 6:**
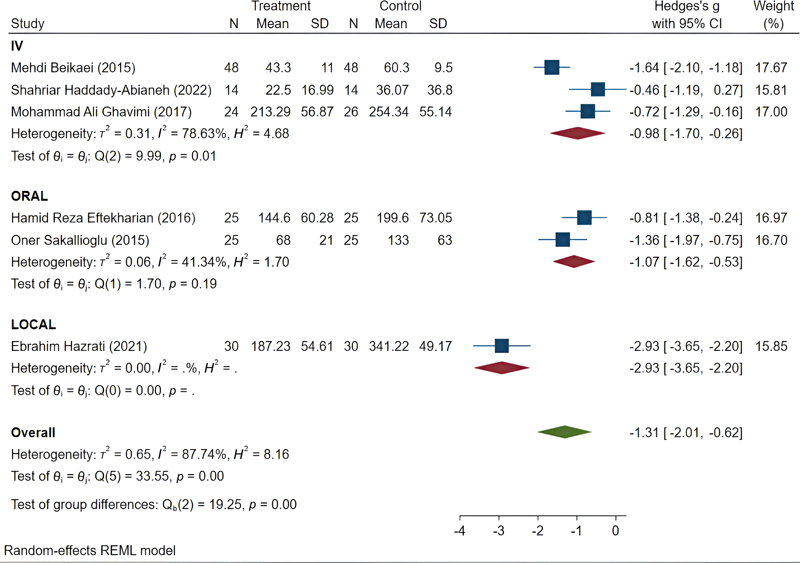
Pooled results of tranexamic acid. CI, confidence interval; SD, standard deviation.

**Fig. 7:**
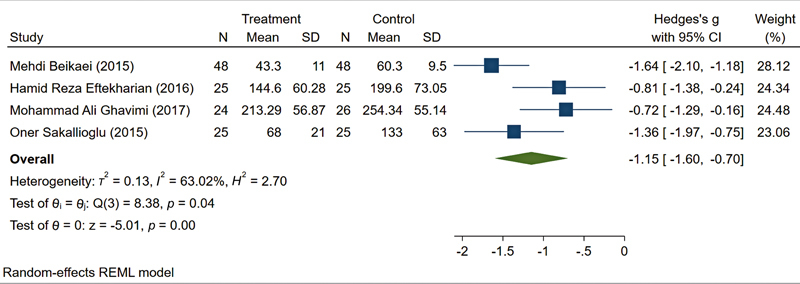
Sensitivity analysis: Effect of excluding high-bias studies on heterogeneity. CI, confidence interval; SD, standard deviation.

The TXA subgroup included six studies, with an overall SMD of −1.31 (95% CI: −2.01 to −0.62). Significant heterogeneity was observed among these studies ( *I* ^2^ = 87.74%). To explore the heterogeneity based on the method of drug administration, the studies in this subgroup were divided into three categories: Intravenous (three studies), oral (two studies), and topical (one study). The overall SMD for the intravenous administration group was −0.98 (95% CI: −1.70 to −0.26), and for the oral administration group, it was −1.07 (95% CI: −1.62 to −0.53). The heterogeneity among studies in the intravenous group was *I* ^2^ = 78.63%, and in the oral group, it was *I* ^2^ = 41.34% ( Fig. 6 ). A sensitivity analysis excluding two studies with potential biases (Haddady-Abianeh et al, 2022 23 ; small sample size) and (Hazrati et al, 2021 11 ; significant baseline imbalance)—reduced the heterogeneity to *I* ^2^ = 63.02% while maintaining a significant effect size (SMD: −1.15, 95% CI: −1.60 to −0.70; Fig. 7 ).

**Fig. 8:**
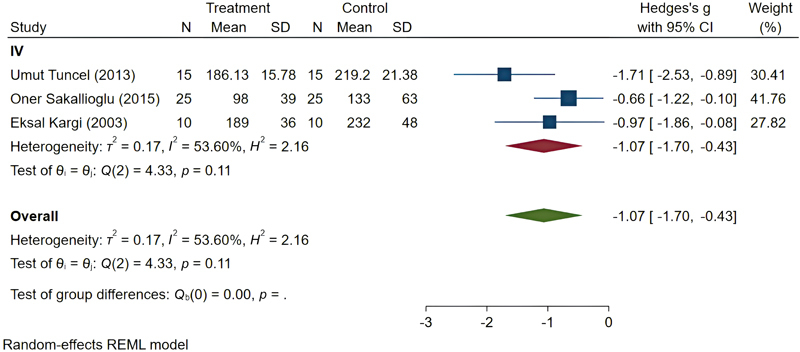
Pooled results of steroids. CI, confidence interval; SD, standard deviation.

The steroid subgroup included three studies, with an overall SMD of −1.07 (95% CI: −1.70 to −0.43). The heterogeneity among studies in this subgroup was *I* ^2^ = 53.60%, which is considered moderate. All included studies in this subgroup administered the drug intravenously ( Fig. 8 ).

**Fig. 9:**
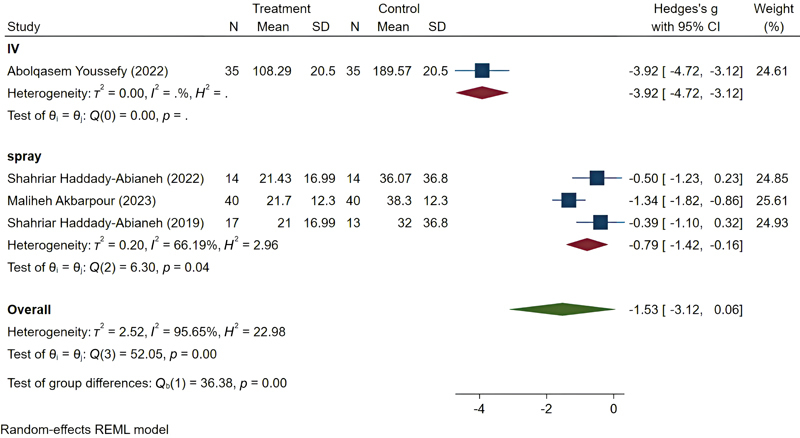
Pooled results of desmopressin. CI, confidence interval; SD, standard deviation.

The desmopressin subgroup included four studies, with an overall SMD of −1.53 (95% CI: −3.12 to 0.06). The heterogeneity among studies in this subgroup was *I* ^2^ = 95.65%, which is very high. To address this, the subgroup's studies were divided into two categories: Intranasal spray (three studies) and IV (one study). The overall SMD for the intranasal spray administration group was −0.79 (95% CI: −1.42 to −0.16). The heterogeneity among studies in the intranasal spray subgroup was *I* ^2^ = 66.19%, indicating relatively high heterogeneity ( Fig. 9 ).

**Fig. 10:**
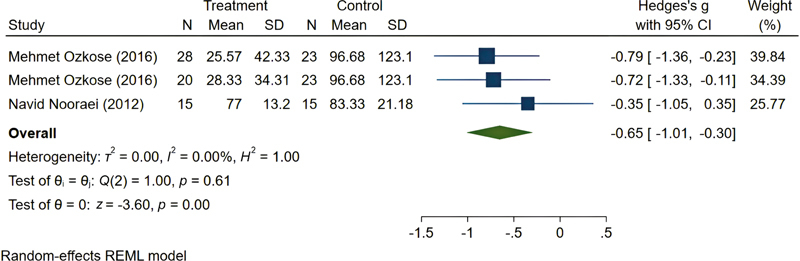
Pooled results of reverse Trendelenburg position. CI, confidence interval; SD, standard deviation.

The RTP subgroup included two studies (three comparisons), with an overall SMD of −0.65 (95% CI: −1.01 to −0.30). The studies in this subgroup were completely homogeneous ( Fig. 10 ).

Additionally, the treatment groups for remifentanil, magnesium sulfate, and clonidine were not analyzed as subgroups because each was represented by only one study in the meta-analysis.

## Discussion

This systematic review and meta-analysis aimed to evaluate the effectiveness of various methods for controlling bleeding during rhinoplasty and septorhinoplasty. Based on the analysis of included studies, various bleeding control methods, including TXA, desmopressin, steroids, magnesium sulfate, remifentanil, clonidine, and the RTP, were evaluated. The results of this study showed that all the mentioned methods, except for remifentanil and desmopressin, had a significant effect on reducing intraoperative bleeding. Below, the findings related to each subgroup are discussed separately.

### Tranexamic Acid

The mechanism of action of TXA involves inhibiting plasmin activity and preventing fibrin degradation, thereby reducing bleeding. This study demonstrated that the use of TXA in rhinoplasty and septorhinoplasty significantly reduces intraoperative bleeding. According to the obtained results, TXA has a significant effect on reducing bleeding, as determined by Cohen's criteria. Previous studies have also confirmed the positive impact of TXA on lowering bleeding. For example, Beikaei et al (2015) and Eftekharian and Rajabzadeh (2016) demonstrated that TXA significantly reduces intraoperative bleeding. 21 25 Additionally, Khajuria et al (2024) conducted a meta-analysis and systematic review to investigate the effect of TXA on bleeding during rhinoplasty and septoplasty, concluding that this drug has a significant impact on intraoperative bleeding. 34 In a systematic review, El Abd et al (2025) found that administering TXA during liposuction can significantly reduce intraoperative bleeding. 35 Similarly, in their meta-analysis, Zhao et al (2019) showed that TXA effectively reduces bleeding during orthognathic surgeries. 36

The stability of results in the analysis of various subgroups based on administration methods (oral, intravenous, and topical) also confirms the potential of this drug as a hemostatic agent. However, further studies are needed to assess the safety of each of these administration methods, particularly regarding the risk of thromboembolism, so that surgeons can effectively reduce bleeding while preventing unwanted complications for patients.

Clinically, the results of the aggregate studies indicate that the use of this drug significantly reduces surgical time, which offers substantial benefits for both the patient and the surgeon. Reducing bleeding not only improves surgical conditions but also enables the surgeon to operate in a cleaner environment with better visibility of the surgical area. These factors, in turn, can lead to improved overall surgical outcomes and increased patient safety.

A significant level of heterogeneity in TXA studies remained even after sensitivity analysis and subgrouping based on the method of administration (except for the oral group, with *I* ^2^ = 41.34%), which is considered an important limitation. Although various analyses confirmed the clinical efficacy of TXA, the interpretation of these results should be approached with caution. For instance, the differences in surgical techniques among the studies—such as the use of closed rhinoplasty in the study by Ghavimi et al (2017) 4 versus open methods in the studies by Beikaei et al (2015) 21 and Eftekharian and Rajabzadeh (2016) 25 —may be one of the key factors influencing this heterogeneity. Additionally, differences in sample sizes and the male-to-female ratio among participants in the studies could be another reason for this heterogeneity, which, although adjusted as much as possible through the analyses conducted in this study, remains a concern.

### Steroids

The primary mechanism by which steroids reduce intraoperative bleeding is through the reduction of inflammation and edema. These effects indirectly reduce bleeding by improving hemodynamic conditions, enhancing surgical visibility, and reducing the need for frequent suction. According to the obtained results, steroids have a significant effect on reducing bleeding based on Cohen's criteria. The studies by Sakallioglu et al (2015) and Tuncel et al (2013) demonstrated that dexamethasone significantly reduces bleeding. 28 29 In their meta-analysis, Hwang et al (2016) found that steroid administration significantly reduces bleeding during endoscopic sinus surgery. 37 In contrast, Koc et al (2011) reported that methylprednisolone does not considerably affect bleeding during rhinoplasty. 38 However, this study was excluded from our meta-analysis due to unclear methods for measuring bleeding. This difference in results suggests that the type of steroid administered and the dosage used can have varying effects on reducing bleeding. Therefore, standardizing these factors in future studies is essential. Additionally, the appropriate timing for administration has been mentioned in the studies, suggesting that it should be given shortly before the start of surgery and also prior to osteotomy.

### Desmopressin

The mechanism of action of desmopressin in reducing bleeding involves increasing platelet activity and coagulation factors. Although the estimated effect suggests a potential impact of desmopressin, the wide CI indicates a lack of certainty, and this ambiguity may stem from the small number of studies. Akbarpour et al (2023) found that desmopressin significantly reduces bleeding in patients undergoing septorhinoplasty. 26 In their meta-analysis, Kim et al (2024) concluded that desmopressin administration can dramatically reduce bleeding during rhinoplasty, which aligns with the findings of this study. 39 In contrast, Leino et al (2010) reported that desmopressin does not significantly affect bleeding in patients with rheumatoid arthritis undergoing hip replacement surgery. 40 This contradiction in results may be due to differences in the type of surgery, as the type of surgery affects the amount of blood loss. Additionally, the target populations differ from one another. Rhinoplasty patients are usually younger individuals, while joint replacement patients are typically older adults with chronic diseases that may be associated with coagulation disorders. The drug should ideally be administered 30 minutes to 1 hour before the start of the procedure if used as a nasal spray. Furthermore, in patients with renal failure, it is advisable to use it cautiously due to the risk of hyponatremia.

However, significant heterogeneity was observed among the studies ( *I* ^2^ = 95.65%). To address this, we divided the studies into two subgroups based on the method of administration: Intranasal spray and intravenous. After separating the study by Youssefy et al (2022 27 ; intravenous administration), the heterogeneity in the intranasal spray subgroup decreased to *I* ^2^ = 66.19%. Although this level of heterogeneity is still relatively high, it may be due to differences in sample size, such as in the study by Akbarpour et al (2023) compared with the other two studies. 26

### Position Change

Position change was evaluated as a non-pharmacological method for reducing bleeding. The RTP reduces venous pressure and blood flow to the head and neck, thereby reducing intraoperative bleeding. The observed effect size indicates a clinically significant reduction in intraoperative bleeding. However, the clinical interpretation of these results should be approached with caution, as the upper limit of the range approaches the threshold of minimal clinical significance. Although the analyses suggest that this method is less effective than a drug like TXA, it can be a suitable option in cases where patients have contraindications to antifibrinolytics or other medications. Additionally, this method has the potential to be combined with other pharmacological approaches to enhance efficacy. Therefore, conducting comparative studies in this area is recommended. Ozkose et al (2016) showed that changing the patient's position can help reduce bleeding. 12 Similarly, Iftikhar et al (2021) found that the RTP reduces bleeding during endoscopic sinus surgery. 41 No heterogeneity was observed among the studies in this subgroup.

### Other Methods

Other methods evaluated in this meta-analysis included magnesium sulfate, clonidine, and remifentanil. Due to the limited number of studies, a comprehensive analysis was impossible. However, preliminary results suggest that these methods have a positive effect on reducing bleeding.

### Limitations

This study presents several limitations that should be considered when interpreting the results. First, there is notable heterogeneity in surgical techniques and blood loss measurement methods across the included studies. Differences in invasiveness, surgeon skill level, and measurement accuracy (e.g., suction vs. weighing) may have influenced the reported outcomes. Although a random-effects model was used to account for this variability, future studies should adopt standardized protocols to reduce methodological inconsistencies.

Second, while funnel plot and Egger's test suggest no major publication bias, the limited number of studies in certain subgroups restricts the reliability of these assessments. Moreover, the tendency of journals to publish positive findings may lead to overestimation of effect sizes.

Third, several studies lacked transparency in allocation concealment and blinding, which raises concerns about potential bias. Although subgroup analyses showed consistent effect directions, caution is warranted in interpreting these results—particularly in subgroups with fewer studies or unclear methodological reporting.

Finally, variation in control group types may contribute to further heterogeneity. Despite excluding studies with active interventions in control groups, three distinct control designs remained among the included studies. Sensitivity analyses indicated stable overall results, but future research should aim for more uniform control protocols.

## Conclusion

The findings underscore the relevance of both pharmacological and non-pharmacological strategies in managing intraoperative bleeding during rhinoplasty. Based on the current analysis and within the limitations of this study, TXA and steroids appear to be among the interventions with high potential for effectiveness, as their effect sizes have consistently shown favorable outcomes. However, due to the limited number of studies and the high heterogeneity in existing literature, this conclusion should be interpreted with caution.

This article may serve as a practical clinical reference for surgeons, aiding in the selection of appropriate bleeding control strategies tailored to the patient's condition and the specifics of the surgical procedure. For instance, steroids can be administered prior to osteotomy to reduce bleeding. In cases where patients have contraindications to the medications evaluated in this study, the findings suggest that positional adjustments may offer a viable alternative. Additionally, desmopressin should be administered 30 minutes to 1 hour before surgery (as a nasal spray) to achieve optimal effect. According to current evidence, desmopressin may present a safer profile compared with TXA, though further studies are warranted to confirm this.
